# People that score high on psychopathic traits are less likely to yawn contagiously

**DOI:** 10.1038/s41598-021-03159-1

**Published:** 2021-12-10

**Authors:** Andrew C. Gallup, Mariska E. Kret, Omar Tonsi Eldakar, Julia Folz, Jorg J. M. Massen

**Affiliations:** 1 Psychology and Evolutionary Behavioral Sciences Programs, SUNY Polytechnic Institute, Utica, USA; 2 Cognitive Psychology Unit, Leiden University, 2333 AK Leiden, The Netherlands; 3 Department of Biological Sciences, Nova Southeastern University, Fort Lauderdale, USA; 4 Animal Behaviour and Cognition, Utrecht University, Utrecht, The Netherlands

**Keywords:** Emotion, Social behaviour, Neuroscience, Psychology, Human behaviour

## Abstract

Considerable variation exists in the contagiousness of yawning, and numerous studies have been conducted to investigate the proximate mechanisms involved in this response. Yet, findings within the psychological literature are mixed, with many studies conducted on relatively small and homogeneous samples. Here, we aimed to replicate and extend upon research suggesting a negative relationship between psychopathic traits and yawn contagion in community samples. In the largest study of contagious yawning to date (N = 458), which included both university students and community members from across 50 nationalities, participants completed an online study in which they self-reported on their yawn contagion to a video stimulus and completed four measures of psychopathy: the primary and secondary psychopathy scales from the Levenson Self-Report Psychopathy Scale (LSRPS), the psychopathy construct from the *Dirty Dozen*, and the Psychopathic Personality Traits Scale (PPTS). Results support previous findings in that participants that yawned contagiously tended to score lower on the combined and primary measures of psychopathy. That said, tiredness was the strongest predictor across all models. These findings align with functional accounts of spontaneous and contagious yawning and a generalized impairment in overall patterns of behavioral contagion and biobehavioral synchrony among people high in psychopathic traits.

## Introduction

The automatic and reflexive tendency to yawn in response to sensing the yawns of others, i.e., contagious yawning, is well-documented in humans^1,2^, and has been observed in a growing number of non-human species including non-human great apes^3,4^, dogs^5^, pigs^6^, and birds^7,8^, among others. Unlike spontaneous yawning, which begins in utero^9^, is ubiquitous across vertebrates^10^, and appears to be a human universal^11^, contagious yawning does not emerge until early childhood^12,13^, is limited to social species^14^, and psychological studies reveal considerable variability in the tendency for people to yawn contagiously. In particular, the percentage of participants that yawn in response to video and/or audio stimuli of others yawning typically ranges between 30 and 60% (e.g.^1,2,15–19^).

This variability in yawn contagion has drawn considerable attention over the last two decades (e.g.^2,20,21^), with numerous published works examining factors that contribute to this response. Overall, the primary emphasis of research in this area has been to explore associations between contagious yawning and empathy or emotional contagion (reviewed by^22,23^). The proposed link between contagious yawning and empathic processing originates from a monograph by Lehmann^24^, and has been elaborated more recently in its inclusion in the Perception–Action-Model (PAM) proposed by Preston and de Waal^25,26^. Accordingly, the motor mimicry of yawn contagion results from a perception–action mechanism that permits the rapid synchronization of states between individuals^27^. Extending from the PAM, contagious yawning has been proposed to represent a basic form of emotional contagion, whereby the yawns, and accompanied emotional or mental state of the yawner, are passed on to another individual^22^. Similarly, automatic facial mimicry is hypothesized to enhance emotional recognition^28^, and has been considered to be a critical feature of emotional contagion^29^. While the motor action pattern of yawning is clearly contagious, as referenced by works above, this theory lacks empirical evidence when it comes to the transfer of emotional states during yawn contagion^23^. Moreover, in general, the role of facial mimicry in the transfer of emotional states is not unequivocal^30^. Therefore, this phenomenon might be better explained by simple behavioral contagion or facial mimicry^31–33^. In contrast to emotional contagion, whereby the synchrony of behaviors and emotions are neurologically linked, simple behavioral contagion represents the copying of the behavior itself. Yawn contagion could be adaptive in the absence of emotional coupling in facilitating collective vigilance and coordinated group behavior^23^. Nonetheless, the idea that contagious yawning is perhaps reflective of empathy or emotional contagion has drawn considerable interest and investigation.

Platek et al.^2^ garnered initial support for a connection between contagious yawning and mental state attribution (a form of cognitive empathy) among samples of university students. These authors found that contagious yawning was positively correlated with performance on self-face recognition and faux pas theory of mind tests, and negatively correlated with measures of schizotypal personality traits. Since then, psychological studies examining associations between individual differences on empathy measures and yawn contagion have revealed mixed findings (e.g.^20,21,34–36^), with most studies reporting no clear relationship (reviewed by^23^). For example, in what was the largest study at the time (N = 328), Bartholomew and Cirulli^20^ found highly consistent individual differences in yawn contagion to video stimuli in an online format, but when accounting for the age of the participants, yawn contagion was not related to empathy as measured by the Interpersonal Reactivity Index (IRI)^37^. More recently, however, Franzen et al.^21^ conducted two large laboratory studies (N = 171; N = 333) and found that participants who yawned contagiously reported significantly higher empathy levels as measured by the IRI. Yet, in a smaller study (N = 97) published recently, contagious yawning was unrelated to the total IRI score, or any of the subscales (cognitive: perspective taking and fantasy; affective: empathic concern and personal distress)^19^. In sum, results from studies linking individual differences in yawn contagion to measures of empathy remain inconsistent.

As an indirect measure of empathy, studies have also examined ingroup or familiarity biases in yawn contagion. Observational studies suggest that naturalistic instances of yawn contagion appear to occur more regularly among friends and family compared to acquaintances and strangers^38,39^. Yet, enhanced attention towards familiar individuals and the tendency to avoid the gaze of strangers or less familiar acquaintances, could have contributed to this effect^40^. To date, the only experimental research to examine in-group or familiarity biases in yawn contagion among humans failed to show an effect^41^. Researchers have also explored gender differences in yawn contagion, as there are marked differences in empathy between men and women^42^. In support of this notion, Norscia and colleagues^43^ reanalyzed data from the aforementioned observational study^38^ and found that women yawned contagiously more so than men. These authors have also noted a similar effect for auditory yawn contagion^39^. However, an analysis across the broader literature demonstrated no differences in contagious yawning between men and women^44^.

Another approach to assessing the link between contagious yawning and empathy has been to study clinical populations with deficits in empathy and to assess variability in other psychological attributes predictive of empathic processing in non-clinical populations. This area of research has led to the examination of (1) group differences in contagious yawning among children with autism spectrum disorder (ASD), and (2) correlational studies measuring individual differences in psychopathic traits and the susceptibility to yawn contagiously. Individuals with ASD tend to be characterized by impairments in cognitive empathy or perspective taking (i.e., theory of mind), while psychopathic traits are associated with reduced affective empathy, including diminished primary emotions such as fear and sadness^45^. Interestingly, both ASD and the prevalence of psychopathic traits are more common among males^46,47^. These populations are also of interest to study with regards to the hypothesized functional significance of yawn contagion in promoting collective vigilance and synchronized group behavior/movement^48–53^. Impairments in imitation and joint attention among individuals with ASD diminish cooperation and coordinated actions^54^. Individuals with ASD also show diminished facial mimicry^55,56^ and less synchronization during interpersonal coordination^57^. Similarly, psychopathy is marked by deceit and a lack of cooperation^58^, and individuals that score high on callous-unemotional traits also display reduced facial mimicry^30,59^ and diminished group cohesion^60^. In addition, a general impairment in biobehavioral synchrony has been implicated in psychopathy^61^.

The first such study on contagious yawning involving individuals with ASD was conducted by Senju et al.^62^. Using standard laboratory procedures, i.e., the presentation of video stimuli with people yawning, children with ASD showed diminished yawn contagion—an effect that garnered significant interest in the scientific community and was subsequently replicated by Giganti and Ziello^63^ and Helt and colleagues^64^. While initially taken as strong support for a link between contagious yawning and empathy, further work refined this view showing that the diminished contagion among children with ASD results from reduced attention to yawning stimuli. When instructed to focus their attention on the eyes of the yawning target in the stimuli, individuals with ASD displayed rates of contagious yawning equivalent to aged-matched control samples (e.g.^18,65^). Moreover, when using eye-tracking to confirm that visual attention is allocated to the stimuli during testing, both typically developing children and those with ASD yawn more often to depictions of yawning than to control clips^66^. In another study, Mariscal et al.^67^ measured contagious yawning along with blood oxytocin levels from both children with ASD and those that were typically developing. Again, no group differences in yawn contagion were found, i.e., children with ASD yawned just as often as typically developing children. However, a positive relationship was reported between contagious yawning and oxytocin among children with ASD, while no such relationship was present in the control group. Although links between contagious yawning and oxytocin continue to be discussed^68^, attempts to experimentally manipulate oxytocin through intranasal administration have found no effect on yawn contagion^69,70^.

To date, only two studies have examined the link between contagious yawning and psychopathic traits. Rundle, Vaughn, and Stanford^71^ had university students (N = 135) from the United States complete the Psychopathic Personality Inventory-Revised (PPI-R)^72^ and watch a series of yawning videos to obtain physiologically defined measures of yawn contagion using facial electromyography and galvanic skin response. When comparing participants that did and did not yawn, there was no difference in overall scores on the PPI-R. However, when examining the individual subscales of the PPI-R, which include fearless dominance, self-centered impulsivity, and cold-heartedness, the latter predicted yawn contagion. That is, participants who scored higher on measures of cold-heartedness were less likely to yawn contagiously. Males from the sample (N = 57) also took part in an eyeblink startle paradigm, and consistent with a link to psychopathy, contagious yawning was less common in men with a lower startle response^71^. The negative association between contagious yawning and psychopathic traits was also recently examined by Helt et al.^19^. Drawing from a slightly smaller sample of university students in (N = 97) also from the United States, participants completed the PPI-R, the Autism Spectrum Quotient (AQ), and the IRI (see above). Unlike Rundle et al.^71^, a negative relationship was revealed between the combined PPI-R and video confirmed yawn contagion (yes/no), i.e., participants that scored higher on psychopathic traits were less likely to yawn contagiously, though no analyses were conducted across the subscales of this measure. By employing eye-tracking, the researchers were also able to show that the negative relationship between contagious yawning and scores on the PPI-R was not moderated by visual attention. A similar negative relationship was found between the AQ and contagious yawning, but unlike measures of psychopathy, the relationship between yawn contagion and the AQ was moderated by eye gaze^19^. Lastly, contagious yawning was not significantly correlated with total empathy or any of the subscales of the IRI.

Based on the overall mixed findings within the psychological literature, and the relatively limited investigation into the link between psychopathic traits and yawn contagion in particular, the current study aimed to provide resolution to this anticipated association. In particular, this investigation sought to replicate and extend upon the findings from Rundle et al.^71^ and Helt et al.^19^, but with a larger and more heterogeneous sample of online participants. Four distinct measures of psychopathy were included to assess the generalizability of this association, and given the importance of physiological variables in influencing yawn contagion (e.g.^15,48,73–78^), measures of sleep and fatigue were taken into consideration. Lastly, given recent debates on the importance of attention in contributing to yawn contagion^22,23,34,65^, both objective and subjective measures of attention to the contagious yawning video stimulus were included.

## Results

**Figure 1:**
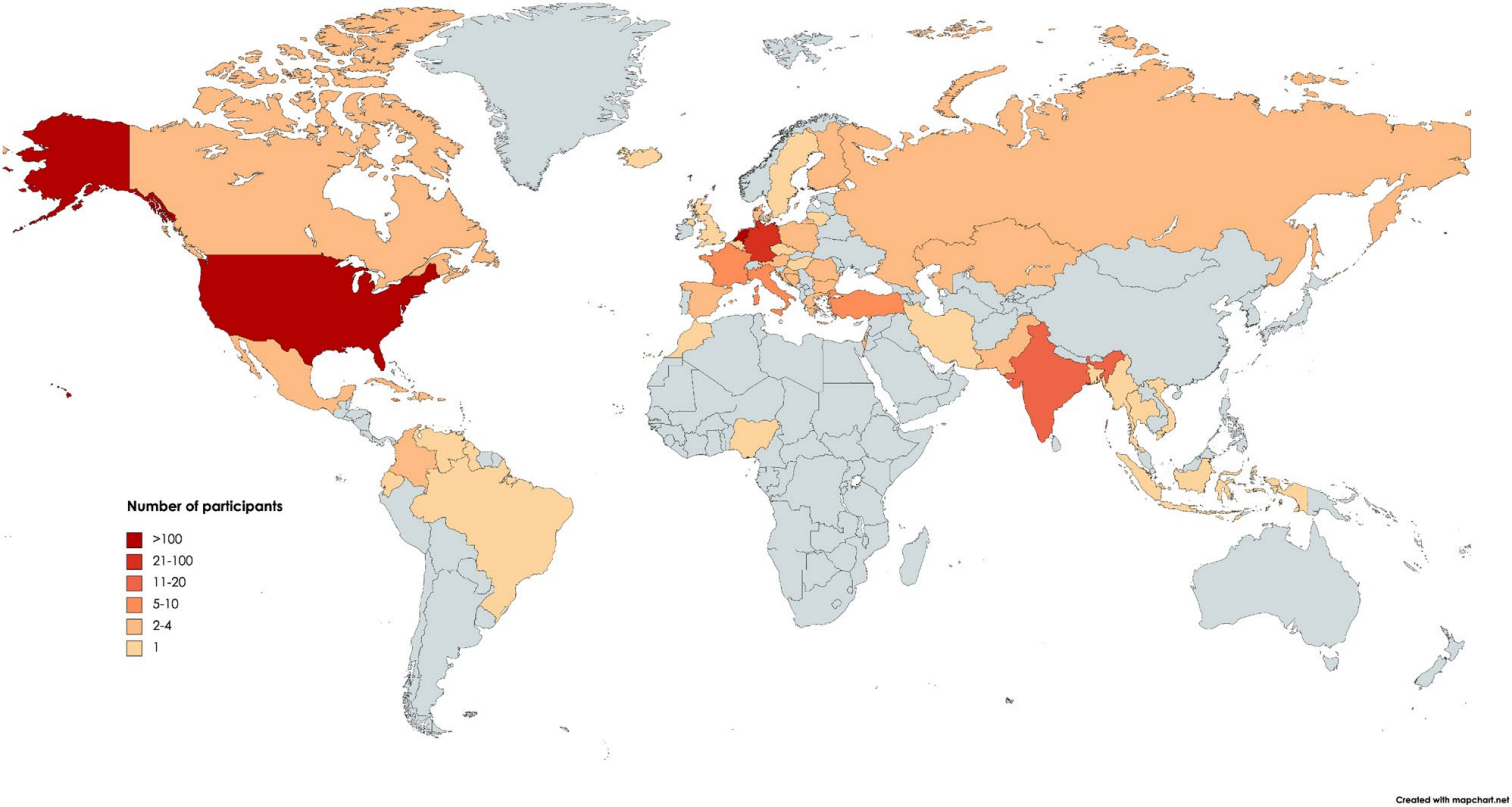
Topographical distribution of sampled participants. Number of participants are depicted in red-scale using different categories (see legend), with an increasing number of participants per country indicated by an increasingly darker color of red. Note that some participants entered double nationalities and that 58 participants entered either a region (e.g. Asia) or ethnicity rather than nationality. This map was created using Mapchart.net.

**Figure 2:**
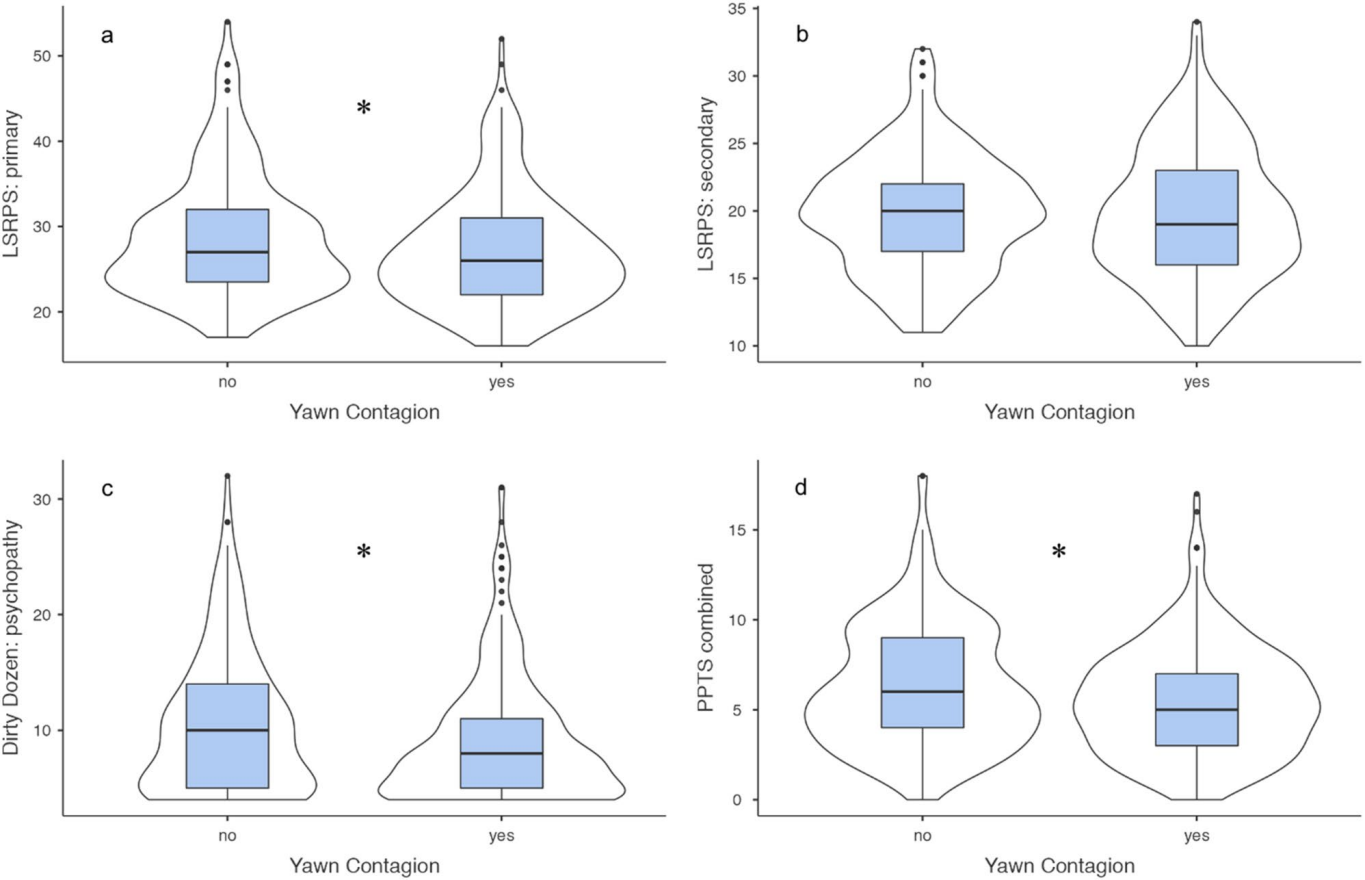
Box and violin plots depicting the relationship between psychopathic trait scores and contagious yawning. Participants that yawned contagiously scored significantly lower (*p < 0.05) on **(a)** the primary psychopathy scale of the LSRPS, **(c)** the psychopathy construct of the *Dirty Dozen*, and **(d)** the combined PPTS. There was no difference between yawners and non-yawners on **(b)** the secondary psychopathy scale of the LSRPS. Box plots represent the median, interquartile ranges, and the whiskers extend 1.5 times the interquartile range for the upper and lower boundary, while the violin plots illustrate the distribution of the psychopathy trait scores.

Across all participants (N = 458), a total of 50 nationalities were identified (see Fig. 1). Descriptive statistics are presented in Table 1. Across the sample, 287 (62.7%) participants reported yawning contagiously in response to the video stimuli. These participants that reported contagious yawning scored significantly lower on three out of the four psychopathy measures (see Fig. 2), including the primary psychopathy scale from the Levenson Self-Report Psychopathy Scale (LSRPS) (*U* = 21,086, *p* = 0.012, Rank biserial correlation = 0.141), the psychopathy construct of the *Dirty Dozen* (*U* = 21,383, *p* = 0.020, Rank biserial correlation = 0.129), and the combined Psychopathic Personality Traits Scale (PPTS) (*U* = 21,453, *p* = 0.024, Rank biserial correlation = 0.126). There was no significant difference in the scores on the secondary psychopathy scale from the LSRPS between those that did and did not yawn (*U* = 24,192, *p* = 0.800, Rank biserial correlation = 0.014).

**Table 1:** Descriptive statistics.

Variable (possible range)	Observed range	*M* ± SD	Skew	Kurtosis
Age in years	18–58	20.42 ± 4.10	4.05	23.6
Prior night sleep in hours	0–14	7.45 ± 1.66	−0.80	3.34
Tiredness scale (0–10)	0–10	4.28 ± 2.40	0.19	−0.59
Objective attention score (0–4)	0–4	2.82 ± 1.02	−0.58	−0.36
Subjective attention score (1–10)	1–10	7.67 ± 2.11	−0.93	0.25
LSRPS: primary (16–64)	16–54	27.70 ± 6.95	0.97	0.86
LSRPS: secondary (10–40)	10–34	19.80 ± 4.63	0.34	−0.25
*Dirty Dozen*: psychopathy (4–36)	4–32	9.64 ± 5.75	1.29	1.40
PPTS combined (0–20)	0–18	5.77 ± 3.20	0.72	0.70

Note: Internal consistency was acceptable for all measures of psychopathy: LSRPS: primary (α = 0.808); LSRPS: secondary (α = 0.671); *Dirty Dozen*: psychopathy (α = 0.770); PPTS combined (α = 0.710).

Subsequent binary logistic regressions were run separately for each psychopathy measure, taking into account participant gender, age, prior sleep the previous night, self-reported tiredness, and objective and subjective levels of attention when viewing the contagious yawning stimulus. In doing so, the primary psychopathy scale of the LSRPS and the combined PPTS remained as significant predictors of yawn contagion (Table 2), i.e., those that scored higher on these measures were less likely to report yawning in response to the video stimulus (*ps* < 0.05). Self-rated tiredness was the best predictor of yawn contagion across all models (*ps* < 0.01), i.e., with yawners reporting higher ratings of tiredness during testing, while none of the following variables were significant predictors in any of the models: gender, age, prior sleep the previous night, and subjective attention. The objective measure of attention was a significant variable in the model assessing the secondary scale of the LSRPS, but not in the expected direction; participants that correctly recalled more details about the video stimulus were less likely to yawn contagiously (*p* = 0.046).

**Table 2:** Output from binary logistic regressions.

Variable	*B*	*p*-value	Odds ratio	Variable	*B*	*p*-value	Odds ratio
Gender	0.021	0.927	1.022	Gender	0.130	0.567	1.139
Age	−0.035	0.156	0.965	Age	−0.027	0.273	0.973
Sleep	−0.014	0.835	0.986	Sleep	−0.014	0.839	0.986
Tiredness	0.144	0.002	1.155	Tiredness	0.144	0.002	1.154
Objective attention	−0.176	0.098	0.839	Objective attention	−0.210	0.046	0.811
Subjective attention	0.033	0.529	1.033	Subjective attention	0.055	0.276	1.057
**LSRPS: primary**	−0.036	0.018	0.965	**LSRPS: secondary**	−0.007	0.767	0.993

Variable	*B*	*p*-value	Odds ratio	Variable	*B*	*p*-value	Odds ratio
Gender	0.047	0.838	1.048	Gender	0.001	0.995	1.001
Age	−0.026	0.284	0.974	Age	−0.027	0.267	0.973
Sleep	−0.017	0.800	0.983	Sleep	−0.014	0.832	0.986
Tiredness	0.145	0.002	1.156	Tiredness	0.135	0.004	1.145
Objective attention	−0.181	0.089	0.834	Objective attention	−0.204	0.053	0.815
Subjective attention	0.046	0.372	1.047	Subjective attention	0.041	0.420	1.042
***Dirty Dozen*: psychopathy**	−0.034	0.058	0.967	**PPTS combined**	−0.067	0.041	0.935

## Discussion

In a large-scale conceptual replication of two previous publications^19,71^, the findings from this study demonstrate a small, but significant negative relationship between yawn contagion and psychopathic traits in a community sample. While these previous studies included a single measure of psychopathy (PPI-R) among university student populations in the United States, the current study assessed the generalizability of this relationship across different scales in a much larger and more heterogenous sample. Consistent with previous reports, we show that participants who failed to show contagious yawning (37.3%) tended to score higher on various measures of psychopathy, including the primary psychopathy scale of the LSRPS^79^, the psychopathy construct of the *Dirty Dozen*^80^, and the combined PPTS^81^. However, the effect sizes from these comparisons were small (Rank biserial correlations = 0.126–0.141). When considering participant gender, age, prior sleep, current tiredness, and objective and subjective measures of attention, only the primary psychopathy scale of the LSRPS and the combined PPTS remained significant, with tiredness of participants serving as the best predictor of yawn contagion across all models. Consistent with a previous review of the literature^44^, and replicating the findings of Rundle et al.^71^, we show no difference in contagious yawning between men and women.

Given that psychopathy is characterized by deficits in emotional empathy, or the ability to be affected by—or share—the emotional states of others^45^, the impetus for examining the connection between psychopathic traits and contagious yawning has focused on purported links between contagious yawning and empathy^19,71^. In particular, Rundle et al.^71^ found that scores on the social-emotional component of the PPR-I, i.e., cold-heartedness, negatively predicted yawn contagion. Since cold-heartedness represents the inconsideration of the emotional state of others^72^, this result was consistent with the notion that contagious yawning represents a basic form of emotional contagion^22,24–26^. In line this view, Helt et al.^19^ found an inverse relationship between combined PPR-I scores and yawn contagion, and that the overall measure of psychopathy was negatively correlated with personal distress in social interactions. Based on these findings, the authors proposed that callous and unemotional traits producing less personal distress might contribute to the inability to achieve bodily resonance with others, and that diminished yawn contagion is due to a malfunctioning of the empathic system.

With regards to the current study, the primary psychopathy scale of the LSRPS—which was the best predictor of contagious yawning from all psychopathy measures—captures indices of individual callousness, egocentricity, manipulation, selfishness, and deceit^79^, and is thus in line with these previous reports. The four questions within the psychopathy construct of the *Dirty Dozen* measure these dimensions as well^80^, and individuals that scored higher on this instrument were also less likely to yawn contagiously. In addition, the results revealed a significant negative relationship to the combined PPTS, which also emphasizes primary psychopathic personality characteristics^81^. Low internal consistency precluded the assessment of the affective responsiveness subscale of the PPTS, which is most closely related to the cold-heartedness subfactor of the PPI-R found to be significant in Rundle et al.^71^. However, the results did reveal a significant negative relationship to the combined PPTS. The secondary psychopathy measure of the LSRPS was the only instrument unrelated to yawn contagion. Unlike the other measures, secondary psychopathy is associated with antisociality, anxiety-driven impulsivity, and irresponsibility, and reflects more of an antisocial-behavioral dimension of psychopathy^82^. In particular, the PPTS was designed with the intention to place a greater emphasis on personality rather than behavior^81^. Thus, yawn contagion appears to be largely associated with affective-interpersonal personality characteristics.

These findings are consistent with the view that contagious yawning is associated with empathy or emotional contagion^22^. In line with this perspective, and as it relates to the current findings, prior works have shown that adolescent males that score high on callous-unemotional traits both score lower on self-report empathy and have reduced facial mimicry, as measured by electromyography, to empathy-inducing video clips^59^ and dynamic emotional expressions^30^. However, it is important to acknowledge that psychopathic traits only represent an indirect measure of empathy, such that yawn contagion could merely reflect a simple feature of behavioral contagion or facial mimicry^31–33^. While empathy represents a superordinate category of PAM^25,26^, features explained by this model, and subclasses of phenomena with the same mechanism, such as imitation and state matching, do not require empathy. This is similar to the Russian-doll model for the evolution of empathy, whereby components of empathy are layered on top of and dependent upon one another^27^. In this case, motor mimicry is at the core within a perception–action mechanism, but in and of itself does not reflect empathy.

Therefore, as an alternative interpretation, the characteristic features of psychopathy might represent an impairment in attachment, social affiliative behaviors, and social connectedness that affects more general features of behavioral contagion^61^. Consistent with the view, Helt et al.^19^ found a similar negative relationship between psychopathic traits and itch contagion, which is not implicated in empathic processing. In addition, a growing number of studies have found that yawn contagion does not correlate significantly with measures of empathy^20,35,36^. In an attempt to delineate between the role of emotional and behavioral contagion in the contagious yawning reflex, Chan and Tseng^34^ compared the effects of empathy, emotional processing, and detection sensitivity to yawning in typically developing populations, finding that only the latter predicts contagious yawning. This same study found that people more likely to detect yawning, but not emotional expressions, are most likely to show contagious yawning. As it relates to group dynamics, psychopathy is thought to originate from disrupted biobehavioral synchrony^61^, which represents the coordination of biological and behavioral processes during social contact and is considered critical for attachment, affiliative bonds, and promoting survival activities in groups^83^. Thus, the reduced tendency to yawn contagiously among individuals scoring high in psychopathic traits could reflect a generalized impairment in biobehavioral synchrony. Moreover, it has been theorized that contagious yawning evolved to enhance collective vigilance^48,49^ and coordinate or synchronize group behavior^50–53^, and these functional accounts for yawn contagion can explain the negative association between contagious yawning and psychopathy in the absence of emotional contagion. For example, groups characterized by higher levels of psychopathy have previously been shown to have more dysfunctional interactions and lower levels of cohesion^60^.

An often-neglected area of study when examining variation in contagious yawning concerns underlying physiologic and circadian factors that contribute to non-social (i.e., spontaneous) yawning. Spontaneous yawns represent the primitive feature of this stereotyped action pattern, and are physiologically driven. Contagious yawning, however, represents a derived characteristic that has appeared more recently among a select group of social species^14^. Since the action patterns of both yawn types appear indistinguishable, the neurological mechanisms governing yawn contagion are likely mediated by the same physiological variables affecting patterns of spontaneous yawning. In support of this view, and consistent with the hypothesis that the motor action pattern of yawning evolved to increase arousal and state change^1,84^ via intracranial circulation and brain cooling^85,86^, socially-elicited forms of contagious yawning can be modulated by different methods of breathing^48^, time of day^74^, cooling/heating to the surface of forehead and neck^48,78^, chewing on gum^76^, ambient temperature variation and seasonal climactic conditions^15,73,77^, and acute physical stress^75^. Collectively these studies support the view that spontaneous and contagious forms of yawning share fundamental mechanistic pathways^87^. In line with this perspective, previous research has shown that both spontaneous and contagious forms of yawning are positively correlated with subjective ratings of sleepiness^74,88^, and the best predictor of contagious yawning here was self-reported tiredness during testing. Thus, when assessing variation in yawn contagion, we propose more attention should be given to a combination of both psychological traits as well as internal physiologic states. Franzen et al.^21^ provides a good example of this approach, measuring pulse rate as an indicator of sleepiness during contagious yawning trials.

With regards to the overall levels of psychopathy reported in this study, the mean scores and measures of variability are similar to previous community samples^79,80^. Previous studies have also used these instruments within incarcerated populations. In comparison to a sample of male inmates in a minimum-security prison, the current levels on the LRSPS were appreciably lower, particularly for the primary measure^89^. In a separate study^90^, which used a different Likert scale, psychopathy levels on the *Dirty Dozen* were also significantly higher among an incarcerated population compared to a community sample. The PPTS was designed to measure psychopathic personality regardless of respondents' criminal history, but was initially measured in two incarcerated samples^81,91^. In this case, the levels in these previous studies were quite similar to those reported here. Thus, future research could examine whether the negative association between psychopathy and contagious yawning would replicate in clinical or incarcerated populations.

While the advantages to this study include the large sample size and relatively diverse composition, there remain limitations. The reliance on self-reported contagious yawning, rather than recording physiologic- or video-confirmed instances of yawning, as was the case in Rundle et al.^71^ and Helt et al.^19^, is a notable limitation. Unfortunately, the Covid-19 pandemic dictated the online format for this study, and prevented the possibility to obtain other measures of yawn contagion. That said, there are two reasons to remain confident in the findings presented here. First, the current results largely replicate earlier studies, an effect not expected if we had obtained inaccurate measures of contagious yawning. Second, prior research has thoroughly demonstrated that self-report is a valid measure of contagious yawning based on substantial agreement between self-report and video-confirmed yawns^41,69,92^. That said, only a binary measure of contagious yawning was assessed, and capturing self-reported yawn frequency could have provided an important source of variability. Another limitation to this study was the lack of a non-yawning control condition. As a result, we were unable to distinguish between spontaneous yawns that could have been reported as contagious. However, we do not view this as a major concern given prior research has shown a very low rate of spontaneous yawning in response to control stimuli (3.3%)^21^. Overall, this study also shares limitations with most research on contagious yawning with regards to the external validity of the contagion stimuli^93^. In this case, participants were shown 50 consecutive yawning clips in short duration and without sound. Thus, in the future, researchers could work to develop and incorporate more ecologically valid stimuli for contagious yawning research.

Our measures of attention were also a limitation. We found no relationship between contagious yawning and the subjective measure of attention to the video stimulus, and an unexpected inverse association between contagious yawning and the objective measure of attention. That is, participants that correctly recalled more details from the video were less likely to report contagious yawning. These findings are difficult to reconcile with previous studies showing a significant effect of visual attention to yawning stimuli on yawn contagion^19,65^. It is possible that rather than providing an index of attention towards yawning, as it was intended, our measure of objective attention inadvertently captured participant attention towards other aspects of the video, such as the running tally of the total number of yawns in the upper-righthand portion of the screen. Thus, participants scoring higher on this measure may have been more attuned to outside features and actually spent less time focused on yawns, which could explain the pattern of results here. Given these limitations, and the continued debate over the roles of visual attention compared to social-connectedness in contributing to contagious yawning^22,34,40,65^, it is recommended that future studies use eye-tracking to assess visual attention to different features of the yawning stimulus^19^.

In summary, the current results, which to our knowledge come from the largest and most diverse sample in the study of contagious yawning to date, show a small, but significant negative relationship between psychopathic traits and the susceptibility to yawn contagiously. Limitations notwithstanding, this study provides a conceptual replication of previous research using multiple measures of psychopathy, suggesting that variability along this dimension is a true and reliable contributor to individual differences in contagious yawning in the general population. While these findings are consistent with an indirect link between contagious yawning and empathy, they also align with functional accounts of spontaneous and contagious yawning and a generalized impairment in overall patterns of behavioral contagion among people high in psychopathic traits^19^. Given the mixed and inconsistent psychological literature on contagious yawning, we hope this research spurs further replication efforts within this area.

## Methods

### Participants

This study included two separate but simultaneous online recruitment methods occurring from September 2020 to May 2021. The first consisted of a university sample drawn from a psychology pool at a public research university in the United States, and the sample size (N = 93) was determined by the total recruitment from a single academic year. The second consisted of a more diverse sample including both university students recruited from a psychology research pool and biology courses in The Netherlands and participants from the community that were recruited through snowball sampling, and the sample size (N = 419) was determined by the total recruitment from a single academic term with a cap set at 425. Participants either received university course research credit or elected to complete the study on a voluntary basis.

Of the 512 total respondents to the online study, nine participants did not indicate that they were 18 years or older, and thus were removed from the analysis. In addition, 41 participants failed to complete the entire study after it was launched, and four participants disclosed that they did not watch any of the contagious yawning stimulus (see below). This left a total of 458 participants (329 female, 124 male, and 5 did not indicate their gender; age *M* ± SD: 20.42 ± 4.10) that were included in the analysis. Across this final sample, a total of 50 nationalities were represented (Fig. 1), though the majority came from the United States and the Netherlands (60.9% combined). The study was conducted in accordance with human ethics guidelines and approved by the Institutional Review Board at SUNY Polytechnic Institute (#IRB-2020-13) and the Psychology Research Ethics Committee at Leiden University (#2021-03-22-M.E. Kret-V2-3088), and all participants provided informed consent prior to partaking in this study.

### Procedure

Participants were provided links to the study offered either through Google Forms or Qualtrics. Respondents first provided their age in years, gender (male, female, prefer not to say), nationality, number of hours slept the previous night, and how tired they were on an 11-point scale (0—not at all tired; 10—very tired). Next, participants were instructed to pay close attention to a 3 min 34 s contagious yawning stimulus derived from online sources (“Yawn-o-meter”). This stimulus presented a compilation of video clips depicting 50 yawns, which were labeled #1–50 on the top righthand portion of the screen, and included 49 yawns from humans and 1 yawn from a dog. The video clips averaged just over 4 s in length and were displayed consecutively without delay. All clips began with either the start of a yawn or mid-yawn prior to the peak muscular contraction^94^, and each clip ended with the conclusion of the yawning act. Only minimal pre- and post-yawn time was shown. The stimulus did not contain any audio, and participants were instructed to watch the entire video prior to moving forward. Given that prior work has shown that self-report is a valid measure of contagious yawning^41,69,92,^ participants were then asked to indicate whether they yawned while watching the video (yes/no).

Participants then filled out five questions assessing their attention to the video while it was displayed. The first four questions were based on their memory of the specifics of the video content, and included (1) recalling how many yawns were presented, (2) remembering whether any non-human animals yawned during the video (yes/no), and (3–4) separately identifying whether two individuals, depicted by still image headshots, were in the video (the first person was from the video, while the second person was not). Based on the number of correct responses to these questions, a total objective attention score was derived (0–4). Lastly, a subjective measure of attention was included, where participants reported how much attention they paid to the video while it was playing on an 11-point scale (0–10; 0–100%). Those that responded with 0 (N = 4) were removed from the analysis.

Next, participants completed the following psychopathic trait instruments for community samples: the LSRPS, which included both primary and secondary measures of psychopathy^79^, the *Dirty Dozen*^80^, and the PPTS^81^. For the LSRPS, participants responded to how much they agreed to 26 statements on a 4-point scale (1—strongly disagree; 4 strongly agree). This instrument was designed to capture indices of both primary psychopathy (16 questions) and secondary psychopathy (10 questions), which was based on a model proposed by Karpman^95,96^. The primary psychopathy scale includes measures of callousness, manipulation, selfishness and deceit, while the secondary psychopathy scale measures antisocial behavior characterized by emotional disorder and impulsivity^65^. Strengths to this measure include the primary and secondary psychopathy distinction, which also differ in terms of affective-interpersonal (primary) and antisocial-behavioral (secondary) dimensions. Internal consistency was high for the primary scale (α = 0.808) and acceptable for the secondary scale (α = 0.671). For the *Dirty Dozen*, participants responded to how much they agreed to 12 statements on a 9-point scale (1—disagree strongly; 9 agree strongly). This instrument was assembled to represent a concise measure of the dark triad, including separate constructs (four questions each) for psychopathy, Machiavellianism, and narcissism. Thus, the brevity is both a strength and weakness to this measure, as it can be easily assessed but is limited in scope. For the purpose of this study, the internal consistency for psychopathy was assessed and found to be acceptable (α = 0.770). The PPTS consists of 20 statements, in which participants responded with either agree (1) or disagree (0). A strength to the PPTS is that it was designed to measure psychopathic personality traits regardless of respondents' cultural background or criminal history^81^. The PPTS was designed to measure four separate subscales of psychopathy, including affective responsiveness, cognitive responsiveness, interpersonal manipulation, and egocentricity. The internal consistency was acceptable for the combined measure (α = 0.710) and interpersonal manipulation subscale (α = 0.660), but low for affective responsiveness (α = 0.586), cognitive responsiveness (α = 0.533), and egocentricity (α = 0.398). Thus, only the combined PPTS score was included in the subsequent analyses. As a validation, all four measures of psychopathy were highly correlated with one another (*ps* < 0.001), with the secondary psychopathy scale from the LSRPS showing the weakest relationship to the others (supplemental material: Table S1).

### Analysis

To assess the association between yawn contagion and psychopathic traits, we initially ran comparisons between contagious yawners and non-yawners across each psychopathy measure. Shapiro-Wilks normality tests were run for all variables, and due to the nonnormal distributions of our data (*ps* < 0.001), a nonparametric Mann–Whitney U test was applied. Then, binary logistic regressions (yawn: yes/no) were run separately for each psychopathy measure, taking into account participant gender, age, hours of sleep the previous night, current tiredness, and objective and subjective measures of attention. All analyses were conducted in Jamovi 2.0.1, and consisted of two-tailed tests with the alpha set to 0.05.

### Ethics

The study was conducted in accordance with human ethics guidelines and approved by the Institutional Review Board at SUNY Polytechnic Institute (#IRB-2020-13) and the Psychology Research Ethics Committee at Leiden University (#2021-03-22-M.E. Kret-V2-3088), and all participants provided informed consent prior to partaking in this study.

## Supplementary Information

Supplementary Information.

Supplementary Table S1.

## Data Availability

Data used in the analyses for this paper are available as supplemental material.
