# Supplementary material for: People that score high on psychopathic traits are less likely to yawn contagiously
Source: Sci Rep. 2021 Dec 10;11:23779. doi: 10.1038/s41598-021-03159-1 (PMC8664845; doi:10.1038/s41598-021-03159-1)
Supplement: Supplementary file 2 — Supplementary Table S1. [file 41598_2021_3159_MOESM2_ESM.docx]

**Supplemental Material**

**People that score high on psychopathic traits are less likely to yawn contagiously**

Andrew C. Gallup^1,3*^, Mariska E. Kret^2^, Omar Tonsi Eldakar^3^, Julia Folz^2^, & Jorg J. M. Massen^4*^

^1^Psychology and Evolutionary Behavioral Sciences Programs, SUNY Polytechnic Institute, USA

^2^ Cognitive Psychology Unit, Leiden University, 2333 AK Leiden, The Netherlands

^3^ Department of Biological Sciences, Nova Southeastern University, USA

^4^ Animal Behaviour and Cognition, Utrecht University, The Netherlands

^*^Corresponding authors: [a.c.gallup@gmail.com](mailto:a.c.gallup@gmail.com) and [jorgmassen@gmail.com](mailto:jorgmassen@gmail.com)

This supplemental file contains the following:

1. Table S1. Correlations between scores on psychopathy trait scales

**Table S1.** Spearman rank correlations between scores on psychopathy trait scales

|  | 1. LSRPS: primary | 2. LSRPS: secondary | 3. *Dirty Dozen*: psychopathy | 4. PPTS  combined |
| --- | --- | --- | --- | --- |
| 1. LSRPS: primary | - |  |  |  |
| 2. LSRPS: secondary | 0.345*** | - |  |  |
| 3. *Dirty Dozen:* psychopathy | 0.450*** | 0.332*** | - |  |
| 4. PPTS combined | 0.600*** | 0.361*** | 0.497*** | - |

*** *p* < 0.001
